# Physical Exercise in Managing Takayasu Arteritis Patients Complicated With Cardiovascular Diseases

**DOI:** 10.3389/fcvm.2021.603354

**Published:** 2021-05-12

**Authors:** Yaxin Zhou, Yuan Feng, Wei Zhang, Hongxia Li, Kui Zhang, Zhenbiao Wu

**Affiliations:** ^1^Department of Clinical Immunology, Xijing Hospital, Air Force Medical University (Fourth Military Medical University), Xi'an, China; ^2^Department of Rheumatology and Immunology, Tangdu Hospital, Air Force Medical University (Fourth Military Medical University), Xi'an, China; ^3^Department of Rheumatology and Immunology, Xi'an No.5 Hospital, Xi'an, China; ^4^Department of Rheumatology and Immunology, Air Force Medical Center, Air Force Medical University (Fourth Military Medical University), Beijing, China

**Keywords:** physical exercise, Takayasu arteritis, large-vessel vasculitis, cardiac diseases, exercise prescription

## Abstract

Takayasu arteritis (TA) is a kind of large-vessel vasculitis that mainly affects the aorta and its branches, and the patients are usually women at a relatively young age. The chronic inflammation of arteries in TA patients leads to stenosis, occlusion, dilatation, or aneurysm formation. Patients with TA thereby have a high risk of cardiovascular disease (CVD) complications, which are the most common cause of mortality. This review summarizes the main cardiovascular complications and the risk factors of cardiovascular complications in patients with TA. Here, we discuss the benefits and potential risks of physical exercise in patients with TA and give recommendations about exercise prescription for TA patients to decrease the risks of CVD and facilitate rehabilitation of cardiovascular complications, which might maximally improve the outcomes.

## Introduction

Takayasu arteritis (TA) is a large-vessel vasculitis that mainly affects the aorta and its branches and occurs in women at a young age (1). The chronic inflammation of arteries can lead to stenosis, occlusion, dilatation, or aneurysm formation (1). TA patients have a high risk of developing cardiovascular complications including cardiac valvular abnormalities (1, 2), coronary lesions (3), acute myocardial infarction (AMI) (4), and myocarditis (5). The TA patients complicated with cardiovascular diseases (CVD) are more likely to have poor prognosis than those without such complications (2, 6), which was challenging to their long-term management. In clinical practice, treatment of TA patients with cardiovascular complications mainly depends on medication and surgery. In the past, doctors tend to advise the patients to avoid exercise in case their hearts or arteries could not tolerate the increased cardiac output or blood pressure. Nowadays, the benefits of physical exercise on the patients with CVD have been widely investigated. The current recommendations about exercise for CVD have emphasized its importance in both prevention and rehabilitation of the diseases (7, 8). This article briefly summarizes the previous findings of cardiovascular complications of TA patients and reviews the literatures about the effect of physical exercise on patients with CVD. Due to lack of specific recommendations about exercise for TA patients with or without CVD based on the studies comprising a large cohort, we discuss the effect of exercise on TA patients according to the existing limited reports and prospect that the TA patients with or without cardiac complications could benefit from physical exercise. Nonetheless, the expertise of rheumatologists is required to evaluate the disease and provide an individual exercise prescription for the TA patients.

## Cardiovascular Complications of TA Patients

**Figure 1:**
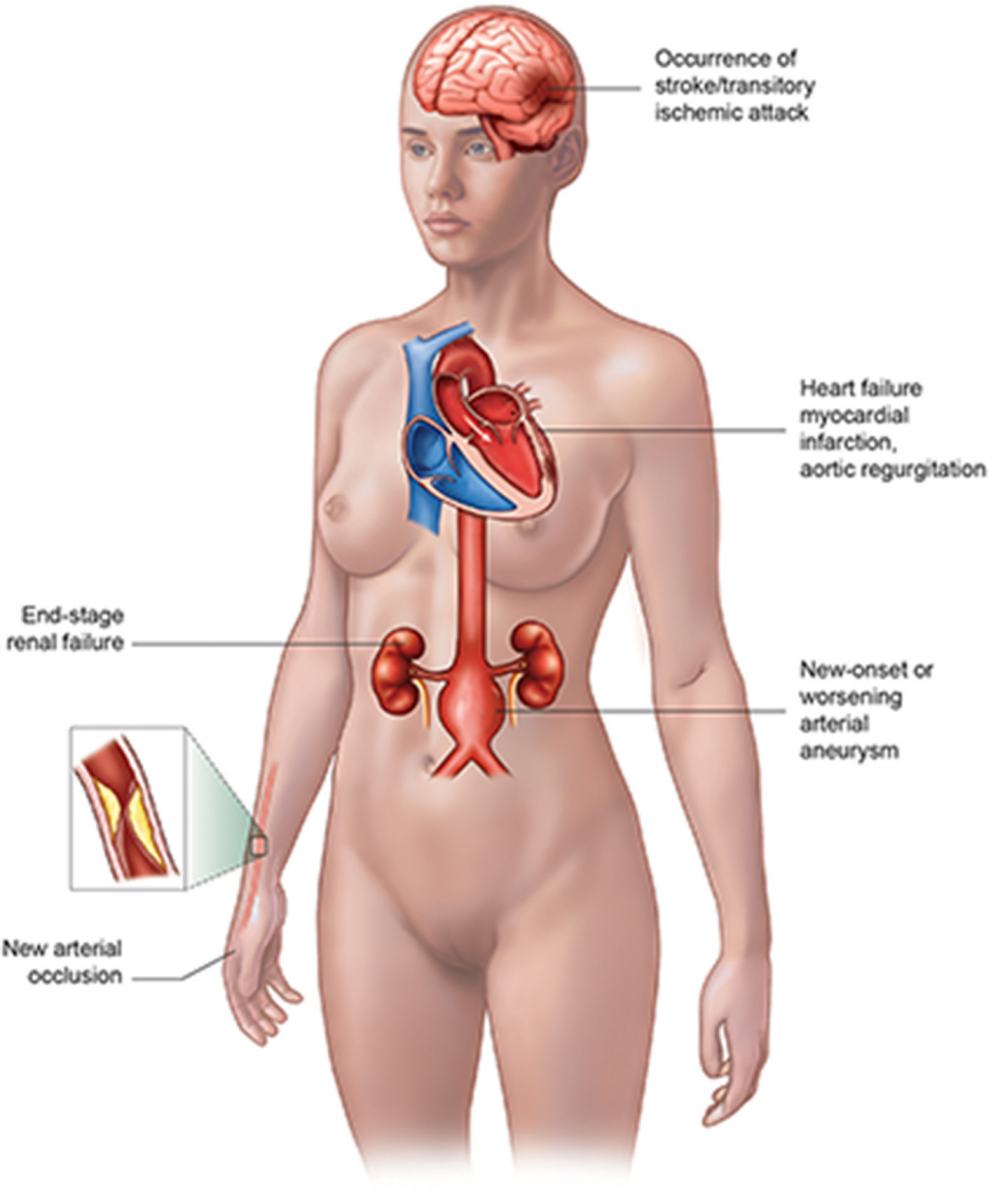
The vascular complications of TA patients.

The vascular complications of TA patients (Figure 1) include myocardial infarction (MI) or heart failure, valvular abnormality, new arterial occlusion especially coronary artery, new-onset or worsening arterial aneurysm, the occurrence of stroke/transitory ischemic attack, and end-stage renal failure (2), where the cardiovascular involvement takes up an important place. The cardiovascular complications of TA patients could be lethal, which might need a medical team consisting of a rheumatologist, cardiologist, interventionist, and other related doctors to rescue the patient's life. Thus, it is extremely necessary for doctors to better understand the cardiac complications of TA patients in order to achieve an earlier diagnosis and better management.

### Cardiac Valvular Involvement

The valvular heart complication secondary to TA is a common event (1, 2), which might result from chronic fibrosis. In a retrospective study of 1,069 patients with disease duration of TA over 25 years, 373 (34.9%) patients had valve regurgitation (9). Among the TA patients with valvular heart disease (VHD), a single valve was involved in over half of the patients and was presented as aortic insufficiency, followed by mitral insufficiency, tricuspid insufficiency, and pulmonary insufficiency (9, 10). The incidence rate of aortic insufficiency was reported as 62.7% (84/134) in China (10), 40.8% (31/76) in Mexico (4), and 44.8% (42/86) in Japan (11). Except for insufficiency, the valvular abnormality in a minority of TA patients might present as stenosis, thickness of aortic valves, and anterior mitral valve leaflet prolapse (10).

Delayed diagnosis and treatment usually occur in TA with valve involvement, possibly because these patients might complain of the same non-specific cardiac symptoms as those with other cardiac diseases (9, 12, 13). Reportedly, more than one-third of the TA patients with valve involvement complained of chest tightness or dyspnea, while the most common symptoms of the patients without valve involvement were dizziness (30.3%) and exertional limb fatigue (25.3%) (9). In terms of pathological characteristics of valves in TA patients, the most common finding was myxomatous degeneration of the aortic valve. Notably, direct infiltration of inflammatory cells into the aortic valve was rarely observed (9, 14, 15). Thus, controlling inflammation is fundamental to both conservative medical treatment and surgical intervention because it can directly or indirectly induce the aortic and valvular lesion (2, 16). In the case of TA patients with valvular involvement, severe aortic regurgitation is a high-risk factor of mortality, necessitating early aortic valve replacement surgery even in very young children. However, surgical reintervention was usually required because of late dilatation of the residual aorta or recurrent aortic regurgitation due to annular dilatation (14, 17). Only when at inactive disease stage, the patients could be prepared to underwent surgical intervention (14, 18).

### Coronary Artery Involvement

Coronary artery involvement is not a rare event in TA patients and could be detected in 10–30% of the patients (3). However, the CT angiography revealed a higher prevalence that 53.2% (59/111) of TA patients had coronary arterial abnormalities (19). The imaging results suggested that most coronary lesions are located in the ostial or proximal coronary artery (20). The three main pathological features of TA patients with coronary involvement are as follows: type 1, stenosis or occlusion of the coronary ostia and the proximal segments of the coronary arteries; type 2, diffuse or focal coronary arteritis, which might extend diffusely to all epicardial branches or may involve focal segments, i.e., skip lesions; and type 3, coronary aneurysms (21). The type 1 lesion is detected most frequently in TA patients (21).

The TA patients with coronary heart disease (CHD) usually suffer prolonged disease (20). However, the presence of cardiac symptoms, disease activity, and other comorbidities did not differ between these patients and other TA patients (19). Among the TA patients with coronary artery involvement, the most common symptom was angina (22, 23), and the age of TA patients presenting cardiac symptoms at onset was younger than that of other patients (23). In the clinical practice, it is challenging to decide on the optimal therapy strategy. Although the symptoms of TA patients could be improved following the glucocorticoid (GC) therapy, the taper of GC often resulted in relapse (24). In addition, the narrowing of coronary arteries might consecutively develop consecutively due to the progression of the inflammatory process. In cases where the patients under conservative therapy showed poor outcome (25), the revascularization should be undertaken promptly. Based on the long-term outcomes of TA patients with coronary involvement, coronary artery bypass grafting (CABG) is superior to percutaneous coronary intervention with stenting (PCI) despite medical therapy (26). PCI had a very high rate of instent restenosis in patients without using corticosteroids so that CABG is the preferred treatment option (27).

### Myocardial Involvement

Generally, acute myocardial infarction (AMI), followed by coronary disease, occurs in the middle or old-age population and is rarely seen in young people. Reportedly, the incidence of AMI in <40 years old is only about 5% in total, and 90% of these cases are males (28). A Japanese research studied the etiology of AMI in young women and found three cases having TA among the 24 female patients <50 years old (29). Therefore, for the young female patients with AMI, systemic disease such as TA should be taken into consideration. Several cases, primarily consisting of young women, have been reported to present AMI as the primary manifestation of TA (30–35). In a 5-year follow-up study, 9% (7/76) TA patients were detected to have AMI through echocardiography (4). Despite its low incidence, myocardial ischemia is one of the major causes of death in TA, with up to 50% mortality in 5 years (4). Prompt and early treatment is essential to the TA patients with AMI. Thus, the medical team should take percutaneous coronary interventions as prioritizing and timely immunosuppressive therapy could improve the cardiac status and long-term outcome (30, 36).

The myocardial involvement of TA patients might also present as myocarditis. A cohort study revealed that myocarditis is a rare and life-threatening presentation of large-vessel vasculitis (36). In a cohort of 139 patients with TA and 24 with giant cell arteritis (GCA), a total of 16 including 14 (10%) with TA and 2 (8.3%) with GCA presented with cardiac failure without a history of ischemic coronary heart disease, and 4/16 patients presented myocarditis as the initial symptom (36). Non-invasive imaging techniques such as transthoracic echocardiography and cardiac magnetic resonance imaging (CMRI) offer an alternative to the gold-standard myocardial biopsy. Myocarditis can result in left ventricular dysfunction and heart failure in some cases with TA (5). Moreover, if the diagnosis of TA with myocarditis is made, steroid pulse, immunosuppressive, and conventional heart failure therapies should be initiated.

## Risk Factors of Cardiovascular Complications in TA Patients

CVD has become the most frequent cause of death in developed countries and China. A large number of studies have identified the risk factors of CVD, which could be categorized as traditional and non-traditional ones. Traditional risk factors for CVD include older age, smoking, high blood pressure, being overweight or obese, diabetes, high cholesterol, and a family history of heart disease, while the non-traditional risk factors include ankle-brachial index (ABI), high-sensitivity C-reactive protein (hsCRP) level, and the coronary artery calcium (CAC) score (37). Table 1 exhibits the recent 10-year findings about the traditional risk factors of CVD for TA patients (38, 40, 42, 43, 45). In addition, some novel risk factors, such as endothelin-1 levels, homocysteine (HCY) levels, erythrocyte sedimentation rate (ESR), and positive lupus anticoagulant, have been identified as risk factors of CVD for TA patients (Table 1) (38, 39, 41, 43, 47). Furthermore, the TA patients who have already experienced cardiovascular complications would be at a high risk to experience CVD recomplications, which alarms the doctors to prevent poor outcomes (44, 46). However, medications or surgical interventions could not ensure that no relapse or complications can occur in TA patients (2). Therefore, physical exercise, as a non-traditional therapeutic method, has become a new research focus to facilitate the management of TA patients.

**Table 1:** Previous findings of cardiovascular risk factors for TA patients.

**References**	**Published year**	**Country**	**Population**	**Cardiovascular complication**	**Risk factors**
(38)	2009	Brazil	22 TA patients and 37 controls	Cardiovascular disease	Hypertension, higher levels of triglycerides, and endothelin-1 levels
(39)	2013	Brazil	29 TA patients and 30 controls	Arterial ischemic events	Homocysteine levels
(40)	2015	China	48 TA patients and 40 age-, sex-, and severity-matched patients with CAD-receiving DES implantation	Major adverse cardiovascular events	Brachial-ankle pulse wave velocity
(41)	2015	UK	22 TA patients	Vascular complications	Positive lupus anticoagulant
(42)	2016	China	60 TA patients with CAD and 60 age- and severity-matched patients with CAD	Major adverse cardiovascular events	High-sensitivity CRP
(43)	2017	USA, Turkey	191 TA patients and 191 controls	Cardiovascular events	SBP, hypertension, CRP, ESR, prior cardiovascular event (cerebrovascular disease, CAD, heart failure), Framingham risk score
(44)	2018	France	17 TA patients who experienced at least 1 stroke and 17 matched TA patients without neurological involvement	Cerebrovascular events	History of stroke
(45)	2019	China	240 TA patients	Cardiovascular events	Brachial-ankle pulse wave velocity
(46)	2019	China	101 childhood TA patients	Vascular complications	BMI level and renal artery involvement
(47)	2020	China	190 TA patients and 154 controls	Coronary artery involvement	Serum HCY and TG levels, TG/HDL-C ratio

*SBP, systolic blood pressure; CAD, coronary artery disease; CRP, C-reactive protein; ESR, erythrocyte sedimentation rate; HCY, homocysteine; TG, triglycerides; HDL-C, high-density lipoprotein cholesterol*.

## Effect of Physical Exercise on CVD

In the past, avoiding physical activity after a cardiovascular event was generally considered. Nowadays, the critical role of physical exercise has been more and more recognized in the integrated management of CVD and the prevention of cardiovascular complications in other diseases (48, 49). First, it needs to clarify the concepts of physical activity and exercise training. The American College of Sports Medicine has defined physical activity as any body movement performed in response to voluntary muscle contraction that increases energy expenditure (50), while exercise training refers to a more elaborated concept, which concerns a planned and structured body movement aimed to improve one or more physical capacities (51). As mentioned above, the CVD complications of TA patients mainly include CHD, AMI, and VHD; here, we discuss the effect of physical exercise on patients with the relative CVD.

In a long-term follow-up of nearly a half million adults in Asia, an inverse association of leisure time physical activity (LTPA) with total mortality was observed among individuals with a severe and often life-threatening disease including CHD and AMI (52, 53). Physiologically, the aerobic exercise could improve blood supply and ventricular function, stabilize the coronary artery clots, and attenuate ventricular remodeling (54). While resistance exercise training in combination with aerobic exercise show benefits on increasing skeletal muscle strength and peak oxygen uptake, decreasing the risks of CVD such as lipid and blood pressure (BP) control and increasing insulin sensitivity (55, 56). In fact, the effect of exercise on patients with CHD is not just positive. Though moderate- to high-intensity exercise, defined as any task requiring ≥5 metabolic equivalent tasks (METs) (1 MET = 3.5 ml of O_2_/min), has been demonstrated to improve the coronary collateral flow and play an important role in cardiac rehabilitation in patients with stable CAD (57), vigorous or extreme endurance exercise is also known to cause or accelerate CAD (58–60). Acute vigorous exercise can transiently increase the risk of AMI and sudden cardiac death (SCD), especially in sedentary patients, but regular vigorous exercise could lower the SCD risk for a long time (61, 62).

For patients with VHD, there have been limited evidences suggesting that regular exercise can control the progression of established VHD. According to published reports, exercise training is thought to be helpful in improving the cardiac function for the VHD patients after surgical intervention (63). For patients with stable asymptomatic moderate VHD, moderate-intensity exercise is possible (64). However, the increased hemodynamic load followed by intensive exercise might raise the pressure on the heart valves and worsen the valvular lesions. Therefore, for patients with VHD, exercise training and continued surveillance are recommended during the post-surgery rehabilitation (63).

## Dose-Dependent Exercise Effect on CVD

Accumulating evidences have supported an inverse dose-response correlation between physical activity levels and mortality (52, 65, 66), and hence, the optimal intensity of physical activity for patients with CVD should be seriously considered. According to a recent study of a large cohort of patients with and without CVD, the patients with CVD might markedly benefit from physical activity to a greater extent than do healthy subjects without CVD. Surprisingly, for the patients with CVD, although the benefit was maximal between 0 and 499 MET-min/week, which was similar to the healthy subjects, the dose-response correlation extended beyond 500–1,000 MET-min/week, and the mortality risk of participants with CVD who performed physical activity ≥1,000 MET-min/week (1,000–1,499 and ≥1,500 MET-min/week) was significantly lower than that in participants free from CVD but had a sedentary lifestyle (67). Since an inverse association of physical activity level with mortality was also observed in patients with CVD, another question is raised whether the very high levels of physical activity exert a protective effect on the cardiovascular system. A recent investigation consisting of 21,758 generally healthy men provided evidence that high levels of physical activity (>3,000 MET-min/week) was associated with prevalent coronary artery calcification (CAC) but was not associated with increased all-cause or CVD mortality after a decade of follow-up, even in the presence of clinically significant CAC levels (68). Meanwhile, the high-volume exercise seems to cause aseptic vascular inflammation and increase the risk of CVD (69). Reportedly, middle-aged men with exercise >2,000 MET-min/week had a higher prevalence of CAC and atherosclerotic plaques but had a more benign composition of plaques, with fewer mixed plaques and more often only calcified plaques (70). However, previous studies supported that exercise could slow the process of atherosclerosis and both high- and moderate-intensity exercises would cause a low incidence of cardiovascular events in a cardiovascular rehabilitation setting among patients with CHD (71, 72).

Oxidative stress reflects an imbalance between production and elimination of reactive oxygen species (ROS) or the repairability of endogenous antioxidant defense system (73). Once the ROS is overproduced and cannot be readily reduced by the antioxidant defense system, the excessive levels of ROS will cause damage to cellular macromolecules such as DNA, lipids, and proteins, eventually leading to necrosis and apoptotic cell death (73). Extremely intense aerobic or anaerobic exercise can induce ROS overproduction and cause skeletal muscle fatigue and cardiomyocyte membrane damage (74, 75). In CVD, oxidative stress is the key physiological process to cause endothelial dysfunction, contributing to increased CVD risk and inflammation (76, 77). Overloaded ROS leads to decreased nitric oxide availability and vasoconstriction, thereby promoting arterial hypertension (76). ROS has also been found to promote atherosclerotic plaque formation and heart fatigue (76, 78). In patients with TA, increased oxidative stress is recognized to participate in the progression of the disease (79). However, regular moderate exercise training decreases oxidative stress and promotes oxidative damage repair (74). Thus, the appropriate exercise intensity for TA should be moderate, and the training should be regular, in case the ROS overproduction aggravates inflammation and results in CVD complications.

## Effect of Physical Exercise on Patients with TA

### Benefits of Exercise Training in TA Patients

Noticing that exercise has become a promising therapeutic tool in rheumatic diseases (80–82), a series of studies has focused on the physical exercise benefits on TA patients. Considering that inflammatory indicators such as serum Interleukin-6 (IL-6), tumor necrosis factor alpha (TNF-α), and CRP have been found to be increased in TA, which were identified as strong independent risk factors for CVD (83), the effect of exercise training on these inflammatory indicators has become the focus for research. Surprisingly, both aerobic exercise and resistance exercise training programs have been demonstrated to diminish the TNF-α and CRP levels in TA patients (84, 85). During acute exercise, skeleton muscles can release IL-6 into circulation (86). However, in TA patients, a sharp increase of IL-6 in response to the acute session of aerobic exercise was attenuated after regular exercise training (84). Biological agents targeting TNF-α and IL-6 have emerged as promising therapeutic options to control refractory activity in TA patients (24). Here, we prospect that the biological agents in combination with exercise training could probably improve the effectiveness of treatments.

The benefits of exercise on TA patients are more than inflammation controlling. It is widely known that metabolic syndrome increases risks of cardiovascular outcomes and mortality (87). In patients with CVD, chronic hyperglycemia caused by insulin resistance triggers oxidative stress of endothelial cells while dyslipidemia contributes to atherosclerotic plaque formation (88). The TA patients have a higher frequency of metabolic syndrome, presenting as hypertension, hyperglycemia, dyslipidemia, and abdominal obesity (89, 90), which are known to associate with CVD and mortality in TA patients (47, 89, 91, 92). Physical activity could modulate the metabolic condition by reducing the triglyceride (TG) levels, low-density lipoprotein (LDH):HDL ratios, and increasing insulin sensitivity (49, 93). Besides, exercise could increase proangiogenic factors, which might be helpful in mitigating the vascular complications of TA patients (84). In a case of arteritis with dyslipidemia, hypertension, and symptoms of claudication in the right leg during walking, the patient showed improved walking capacity and cardiovascular function after 16 weeks of unsupervised exercise training (94). Stefano Lanzi et al. directed a 28-year-old man with TA and symptoms of arterial lower limb claudication to participate in supervised exercise training. Consequently, the patient showed improved walking performance and physical function of the lower extremities when the training was completed (95). Moreover, the increased walking capacity could also improve the social and psychological functions of the TA patients with claudication of the lower limbs (84, 94).

For the TA patients with or without cardiovascular complications, the benefits from physical exercise include multiple aspects: First, regular moderate intensity of exercise attenuates the inflammation by reducing the level of CRP and ESR and cytokine production in TA patients such as TNF and IL-6 (96). Second, physical exercise improves the abnormal metabolic status of TA patients and thus decreases the CVD risk. Third, researches have also shown that exercise could possibly change endothelial function and protect the vascular endothelium of TA patients, which might decrease the incidence of related complications and death (84, 97). Fourth, it is more common for TA patients to have a problem with mental health (98) while LTPA is found to be inversely associated with stress, anxiety, and depression, which is meaningful to improve the quality of life (93). Finally, regarding its economic factor, physical exercise is a cheap therapeutic method that might lessen the financial burden in TA patients.

### Possible Risks of Exercise Training in TA Patients

Although most of the studies support that TA patients could get benefit from exercise to decrease the CVD risk, the potential adverse effect of exercise on TA patients should be seriously taken into consideration. Herein, we discussed the conditions that make exercise prescription unsuitable for TA patients.

Though high-intensity exercise was observed to reverse the pathological remodeling and improve systolic and diastolic blood pressure in patients with CHD (71), other studies have reported that extreme endurance exercise may increase the coronary plaque volume in the individuals with baseline CAD and also increase the CRP and HDL (60). Because high levels of CRP and HDL have been demonstrated to be risk factors for TA patients to develop CVD, vigorous or extreme exercise should be strictly limited for TA patients, especially for those with pre-existing CHD.

In a cohort of 21 patients with connective tissue disease-associated pulmonary arterial hypertension (PAH), the heart rate at rest, peak oxygen consumption, oxygen saturation, and maximal workload, as well as systolic pulmonary artery pressure and diastolic systemic blood pressure were significantly improved after in-hospital exercise training for 3 weeks followed by home-based training for 15 weeks (99). However, considering that reduced stroke volume during exercise is a plausible factor to increase the risk of decompensation for PAH patients (100), light to moderate exercise training practice is more suitable and should be well-supervised.

## Exercise Prescription for TA Patients

### General Management of TA

**Figure 2:**
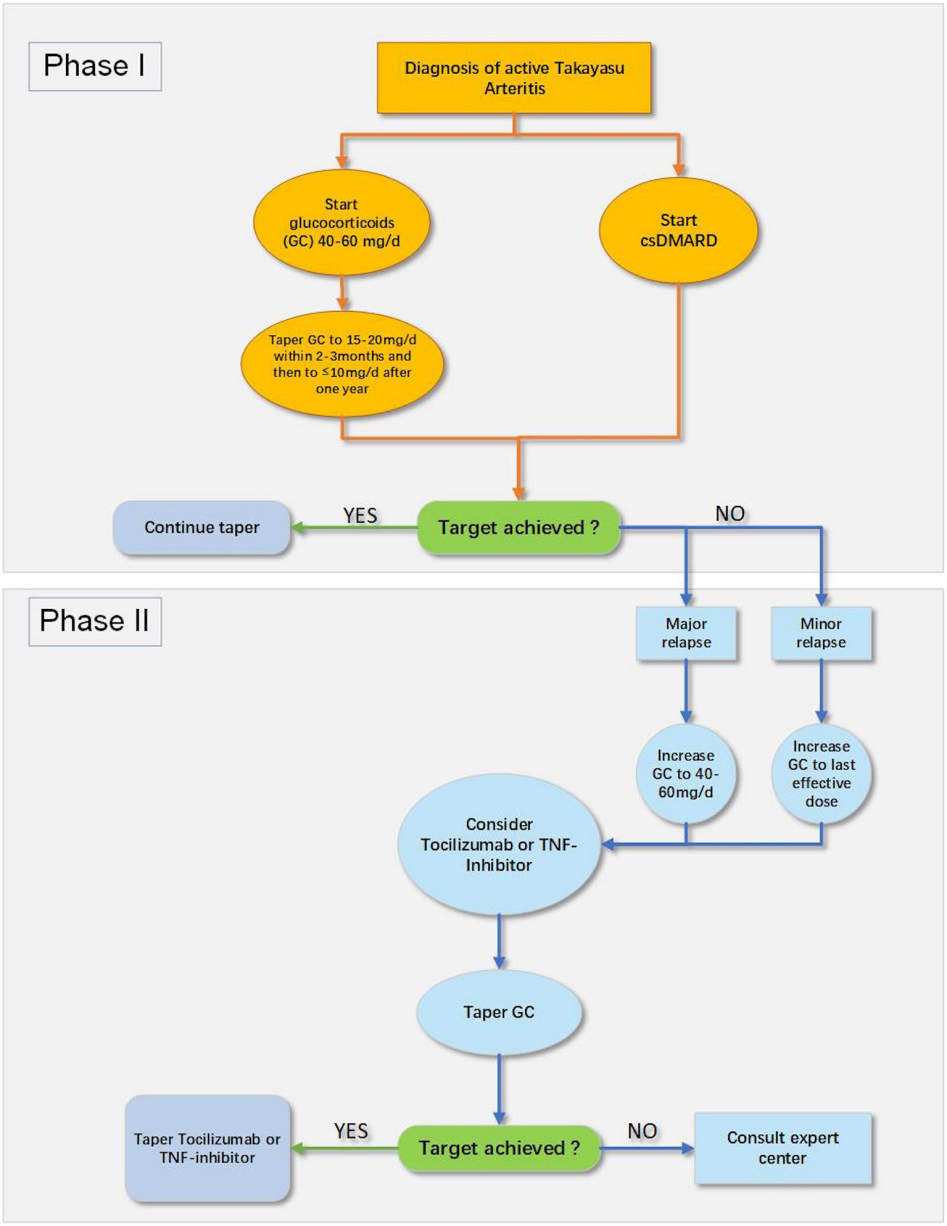
The 2018 EULAR recommendation for pharmacological treatment of Takayasu arteritis (TA) (24). csDMARD, conventional synthetic disease-modifying antirheumatic drug; GC, glucocorticoids; TNF, tumor necrosis factor.

The 2018 European League Against Rheumatism (EULAR) recommendation for pharmacotherapy to TA patients is exhibited in Figure 2 (24). A nationwide study revealed that TA patients were at a high risk of relapse and experienced a vascular complication during the first 10 years following diagnosis (2). Intriguingly, no validated therapeutic strategy is yet available to sustain remission in patients through either conservative therapy or surgical intervention (24). Moreover, TA patients are likely to live a poor quality of life (101), which brings forward higher requirements for doctors to comprehensively manage TA patients. The rheumatologists have to look for novel treatments combined with pharmacotherapy to improve the prognosis of TA patients. This article above has focused on the double-edged effect of physical exercise on the patients with CVD, which raises a challenge to establish a practicable, safe, and effective exercise prescription for TA patients.

### Recommendations About Exercise Prescription for TA Patients

In clinical practice, rheumatologists or cardiologists need to evaluate the physical capacity of TA patients before giving advice on physical exercise. According to several studies, related assessment includes muscle strength tests containing dynamic 1-repetition maximum tests for the leg-press and the bench-press exercises, arm curl (with the dominant arm), and isometric strength (assessed by handgrip, with the dominant arm), muscle function tests (assessed by the TUG and the TST tests) (84), walking capacity [assessed by progressive graded treadmill protocol (102), 6-min walk test (103)], and lower limb function test [assessed by stair-climbing test (104)]. Also, it is advisable to limit the workload during exercise training for TA patients with CVD. The pre-exercise screening using 12-lead electrocardiogram (ECG) is effective in avoiding sudden cardiac death in sports in young athletes (105), which should also be considered to be applicable on TA patients.

With respect to the intensity of exercise, the current guidelines on CVD prevention recommend at least 500–1,000 MET-min/week of moderate-to-vigorous physical activity (7, 106). Although, a recent study supported that patients with CVD could get maximal benefits of the physical activity at 0–499 MET-min/week, which continued above 500–1,000 MET-min/week (67). Nonetheless, the optimal intensity of physical exercise for TA patients remains unknown.

Referring to the existing studies, there are two main patterns of exercise training on patients with TA or arteritis. (1) Supervised exercise training includes aerobic exercise such as treadmill walking, machine-based resistance exercise, or aerobic exercise in combination with resistance exercises (84, 85, 95). In addition, aerobic exercise consists of a 5-min warm-up, followed by 30–50 min of walking training and a 5-min cooling-down period. (2) Unsupervised training pattern. The patients with claudication can be instructed to walk at least 1 h daily until the maximum claudication pain was reached at least five times a week, but the walking speed is dependent on themselves (94). The supervised or unsupervised exercise training finally results in an obvious improvement of physical capacity and cardiac function, and the details are summarized in Table 2.

**Table 2:** Summary of studies about exercise training on patients with arteritis.

**References**	**Arteritis**	**Number of patients receiving exercise training**	**Age (y)**	**Body mass index (kg/m^**2**^)**	**Disease features**	**Drugs**	**Surgical treatment**	**Mode of exercise**	**Duration and frequency of exercise**	**Supervised or unsupervised exercise**	**Effects of exercise training**
(94)	Arteritis with no identifiable cause	1	33	29.4	Hypertension, dyslipidemia, claudication in the right leg	Simvastatin and cilostazol	No	The patient walk at least 1 h daily until the maximum claudication pain. The subject was free to determine walking speed.	16-week, at least five times a week	Unsupervised exercise training, weekly phone calls were made to monitor adherence to training.	↑: Claudication distance and total walking distance during treadmill and 6-min walking test
											↓: SBP, DBP, rate pressure product, and LF/HF ratio
											↑: QoL (assessed with SF-36)
(84)	TA	6	35.3 ± 6.6	26.3 ± 4.6	Claudication of extremities, 4/6; Decreased brachial artery pulse, 6/6; Blood pressure difference >10 mm Hg, 5/6; Bruit over subclavian arteries or aorta, 5/6; Arteriogram abnormality, 6/6	Acetylsalicylic acid, 6/6; Prednisone, 1/6; Azathioprine, 1/6; Methotrexate, 2/6; Mycophenolate mofetil, 1/6; Statins, 1/6	Not mentioned	The training sessions consisted of a 5-min warm-up followed by 30–50 min of treadmill walking, and a 5- min cooling-down period. The walking duration was gradually increased every 4 weeks, from 30 to 50 min. The intensity of the exercise sessions was set at the heart rate correspondent to the interval between the VT and the respiratory compensation point.	12-week, twice-a-week	Supervised exercise training	↑: Muscle strength and physical function
											↑: Time to reach VT
											= V'O_2_ peak, time-to-exhaustion;
											=: Endothelial function
											= QoL (assessed with SF-36 and HAQ
											↓: TNF
											↑: VEGF and PDGF AA
(95)	TA	1	28	17.8	Hypertension, Claudication of lower extremities, thickness of the arterial wall and stenosis/occlusion of digestive, renal, and iliofemoral arterial axes.	Prednisone and methotrexate	No	The training sessions consisted of 36 sessions. Each training session started with a 5–10 min warm-up and ended with a 5-min stretching cool-down period. One session weekly was mainly focused on strengthening of lower limbs and include different type of walking (heel and toe walking, skipping walking, side-to-side walking, power-jogger walking) and resistance exercises focused on the main muscle groups of the lower limbs performed with an elastic band. During the two other weekly sessions, outdoor Nordic walking was performed. Training session duration was progressively increased (from 30 to 55 min) according to patient's tolerance. The intensity was mainly set at 12–14 on the 15-grade Borg scal (moderate intensity).	Over 12 week, 3 times a week	Supervised exercise training	↑: Pain-free walking distance, maximal walking distance, 6-min maximal walking distance;
											↑: Short physical performance battery;
											↓: Stair climbing test
(85)	TA	140	36.6 ± 7.8	23.1 ± 2.4	Increased level of TNFα, CRP and ESR; normal BVAS	Not mentioned	Not mentioned	A complete resistance exercise routine took ~1 h and consisted of eight different progressive machine-based resistance exercises, namely leg curl, leg extension, leg press, seated row, shoulder external and internal rotation, latissimus pull down, butterfly and butterfly reverse, and shoulder extension and flexion. Each exercise was composed of three sets with 8 to 12 repetitions at a weight of 60–80% of one's repetition maximum. If all three sets of an exercise (12 repetitions in total) were completed successfully in three consecutive resistance exercise sessions, the weight would be elevated by at least 5% in the next session.	12 weeks, twice a week	Supervised exercise training	↓: TNFα, CRP, ESR and BVAS

*TA, Takayasu arteritis; SBP, systolic blood pressure; DBP, diastolic blood pressure; LF/HF ratio, ratio of the low- and high-frequency bands in heart rate variability; QoL, quality of life; SF-36, Medical Outcome Study Short-Form 36 General Health Survey; VT, ventilatory threshold; V'O_2peak_, peak oxygen uptake; HAQ, Health Assessment Questionnaire; TNF, tumor necrosis factor; VEGF, vascular endothelial growth factor; CRP, C-reactive protein; ESR, erythrocyte sedimentation rate; BVAS, Birmingham Vascular Activity Score;↑, increase; ↓, decrease; =, similar*.

Considering that the adverse effect of exercise on the cardiovascular system is dose dependent, which is mainly induced by irregular and vigorous exercise, we thus recommend a regular and moderate exercise prescription for TA patients complicated with CVD. However, if the patients are previously sedentary, low-intensity exercise such as walking (2–3 METs) should be preferred when they initiate the exercise training program. The walking speed or intensity of exertion should be increased gradually within about 2–3 months to avoid acute vigorous exercise-associated SCD (107). Except for a careful history and physical examination and evaluation of physical capacity, the disease activity and inflammatory indicators such as ESR and CRP of TA patients should be taken into consideration when giving exercise prescription. If the patients are in active or relapse phase, low-intensity LTPA such as walking slowly or strolling is more recommended in case oxidative stress overproduction aggravates the inflammation and endothelium damage of patients. When patients stay in remission, moderate-intensity exercise is more recommended for TA patients, such as cycling (as transportation), table tennis, and walking briskly. Resistance exercise is also helpful for TA patients in primary and secondary prevention of CVD (56, 85). The exercise training program should last for more than 12 weeks and at least twice a week to achieve the goals of strengthening the muscles, improving the cardiorespiratory fitness, reducing the inflammation, and lowering the CVD risks for TA patients. Besides, during exercise training, the TA patients should be well-supervised by experienced therapists. Once adverse signs and symptoms occur, such as dizziness, excessive shortness of breath, chest pain or pressure, and heart rhythm irregularities, the exercise should be stopped immediately.

## Conclusion

TA is a kind of inflammatory disease mainly affecting arteries, and these patients are in high risk of cardiovascular complications. For the TA patients, physical exercise has double-edged effect on the cardiovascular system. Rheumatologists need to give individual exercise prescriptions to improve the prevention, treatment, and rehabilitation of CVD for TA patients.

## Author Contributions

YZ: conception, design, and drafting the article. YF and KZ: writing and revising the article. WZ: collecting literature and writing the article. HL: writing the article. ZW: writing the article and administrative support. The final manuscript has been approved by all the authors.

## Conflict of Interest

The authors declare that the research was conducted in the absence of any commercial or financial relationships that could be construed as a potential conflict of interest.
